# Some Thoughts on Foot-Sore Horses in Our Cities, with a View to Ameliorate or Prevent the Same1Read before the Pennsylvania State Veterinary Medical Association, March 6, 1901.

**Published:** 1901-07

**Authors:** Charles Williams

**Affiliations:** Philadelphia, Pa.


### SOME THOUGHTS ON FOOT-SORE HORSES IN OUR CITIES, WITH A VIEW TO AMELIORATE OR PREVENT THE SAME.[1]

By Charles Williams, V.M.D.,
PHILADELPHIA, PA.

When asked for a title to my paper by one of the committee I was so undecided which end of the horse to take up that I was entirely unable to name it. But the more I thought on the subject it seemed to me the most prevalent trouble that comes to us city veterinarians would be of interest. I think Percival remarked that navicular disease was the rock on which more good horses are wrecked than on any other.

In a short paper like this it is not my design to go into a detailed account of the numerous distinct diseases of the horse's foot, for that has already been done much more ably by many writers, and I feel sure you are all already only too familiar with them. Neither do I propose to take up any one disease, but I will allude

[1] Read before the Pennsylvania State Veterinary Medical Association, March 6, 1901.

to several in a cursory way. My desire is to offer a few thoughts and ideas which presented themselves as the result of my experience in treating and shoeing foot-sore horses. You all know the foot of the horse consists of a tough, elastic, horny box in the shape of a truncated cone (which may be separated into four separate and distinct parts, namely, sole, frog, wall, and coronary band), inside of which on the under surface of the pedal bone is the velvety tissue, and over the anterior face and sides of the bone is the laminated tissue. These are highly vascular and richly supplied with nerves. The rear and base of the heels are composed of a rather insensible spongy and elastic tissue which, with the underlying frog, is designed to act as buffers to break the jar that must take place with motion.

The horse in nature is unshod, of course, and unused to any kind of restraint, and the foot then comes flatly in contact with the soft earth which causes equal pressure on the walls and frogs together with a gentle but nevertheless firm reinforcing support to the sole as well. When we try to continue this natural state of the foot under our artificial conditions we would have to continue the soft and natural soils in our roads which would soon become impassable or, at any rate, seriously curtail the size of the loads and the speed which our animals now attain. In order that our animals may be of the greatest utility to their owners we must have hard, resisting roads and pavements on which the unshod animals would break away the hoofs till the sensitive tissue is reached and the animal become "sore" (lame). This is the first and most simple form of soreness. In order, therefore, to render our animals useful on these thoroughfares we must have recourse to an artificial protection of iron in different forms and shapes, and oftentimes use it in connection with rubber and other substances to decrease the jar and also to avoid the danger of slipping on the smooth or icy surfaces. The average shoe elevates the foot from the ground to a greater or less degree, which does not allow the frog or sole the support it should receive, and hence all the weight of the animal is brought to bear on the walls, instead of the sole and frog receiving their share, and thus undue strain and jar are received by the sensitive lamina, which produce congestion and often inflammation or laminitis in varying degrees, together with corns at the heels.

Horseshoers in the past, we are told, were very fond of paring away the sole and shaving off the frog till both were seriously reduced. This practice has been so strongly decried that now they have swung around to the other extreme, and now do not even remove the superfluous horn and frog that have failed to be shed normally on account of the protection of the shoe. This becomes extremely hard and dry, because the average stall is drained and dry, and, also, our streets being smooth and well drained, the front feet seldom come in contact with water except when it rains, or possibly, Saturday night when a little flaxseed meal is dampened and applied to the soles, but this soon falls out, and next morning it would hardly be apparent that there had ever been anything like water about the foot. Thus this surplus horn that is now unnourished becomes excessively hard and dry for want of moisture (which in the natural state is provided every night in the dews on the pastures); it then acts as a foreign body which in time will cause the sensitive velvety tissue to become bruised and sore. The point of the frog, in the same way, pressing over the articulation, acts as a stone in the foot, and bruising the underlying parts which are adjacent to the articulation induces navicular arthritis or aggravates a previously existing trouble. Many shoers, to obviate this, thoroughly cover the sole with a copious supply of tar and oakum and then adjust a leather and shoe to hold the same in contact with the sole, thus thoroughly sealing the foot from all influences of air or moisture, and when the shoes are removed again the sole crumbles and is so easily removed that it may almost be scraped out with the back of the knife, but it is dry, hot, and brittle. Now that our streets are largely asphalt, and consequently treacherous and slippery at times, the rubber pad has been devised to obviate this, and it has no doubt done more or less good as well as harm. When properly applied they act as a buffer in the high-acting carriage horses now so much admired, and give frog pressure, hence preventing contraction. But the dangers are that certain ones are constructed with the view only of preventing slipping. They, therefore, give an undue amount of pressure at the toe and also over the point of the frog, and thus produce either navicular disease or aggravate a previously existing though latent trouble.

Anyone who has examined the foot of a horse which has been running to pasture will invariably find the sole moist, for it is in constant contact with the moist earth and grass, and has no chance to dry. Therefore, from this fact I have frequently made the assertion that if the sole is well covered the rest of the foot will take care of itself.

My attention was drawn to the Lackey method of shoeing horses some ten years ago by seeing old sore trotters and others with apparently incurable lameness get well and make useful animals. I was very skeptical at first, but when I saw the results accomplished I was induced to try it on my own horses with most gratifying results, after having tried almost all of the other approved methods of shoeing, such as bars, tips, rubber pads, etc. The system is simply this: a piece of sole-leather reinforced at the heels and riveted firmly and flatly on to the shoe to prevent sagging or bulging; this you will see gives a firm but somewhat elastic support to the sole of the foot. The foot is prepared in the usual way, only care is taken to remove the prominent parts of the bars and point of frog, and the sole is cupped slightly at the toe. The fitted shoe is then placed in position, and one of the toe-nails driven. This insures it against slipping and keeps it in perfect position. The shoe is then pried off at the heel and the sole anointed with a coating of an oily resin soap. A piece of wet sponge is then laid over the frog and bars well back, so as to not cause undue pressure at the point of the frog. The shoe is then brought back in place and the fastening completed. On examination of the sole you will find firm pressure at the heels, and the rest will be soft and elastic over the centre of the foot. Horses shod constantly in this manner retain the softness and toughness of the horn of the foot, which may be pared without difficulty, and the shavings will be tough and elastic. The heels widen and become full and the animal moves with a freedom and a conscious indifference to the kind of road he is likely to encounter, which it is a pleasure to contemplate.

Even cases that have been pronounced hopeless without neurectomy have so far recovered as to be satisfactory without the use of the knife. I have adopted this method for caring for horse's feet that have been laid up by some lameness when the animal uses the foot scarcely at all and the member in consequence begins to dry out and contract, and hence increase (in a mechanical way) the trouble. Sponges tied on to the wall and placed in the sole, thus making a veritable sponge poultice, and kept wet will be found to be more pleasant as well as more efficient than the average poultice.

Before closing this paper I wish to call attention to one more important point in treating and caring for the foot, and that is the proper dressing and proportioning of the same. I believe the out of balance or proportion of the feet of our fast light harness horses has caused more trouble than probably any other one cause. My ideas on foot measurements were first derived from the articles on "Balancing" in the *Chicago Horseman*, and on applying them I

have found them practically correct. The toe in all cases should be just two inches longer than the heel is high in normal feet, but, of course, in diseased or abnormal feet the judgment of the practitioner must come into play. For instance, in chronic laminitis when the pedal bone is dipped and the horse wearing the heels badly will bear the heels much lower than one which is suffering with navicular arthritis. Again, where the heels are very sloping they must be left a little higher in order to preserve the proper angle of the pastern, which should be about 50° in front and 55° back. The measurement of the feet is nearly in direct proportion to the height and size of the animal. Those which I here copy from the *Horseman* are for light harness horses, viz.:

| | | | | | | | | | |
|---|---|---|---|---|---|---|---|---|---|
| Height, | 14.2 | 14.3 | 15.0 | 15.1 | 15.2 | 15.3 | 16.0 | 16.1 | 16.2 and over. |
| Toes, in inches, | 2⅞ | 3.0 | 3⅛ | 3¼ | 3½ | 3⅝ | 3¾ | 3⅞ | 4.0 |
| Heels, in inches, | ⅞ | 1.0 | 1⅛ | 1¼ | 1½ | 1⅝ | 1¾ | 1⅞ | 2.0 |

These measurements are approximate, and will vary with different feet, but they serve as a starting-point from which to calculate our variations, and our judgment must govern. I usually take a pair of compasses and size up my horse and mark the toes and heels, first approximating the rule, then I make a lower mark, say one-eighth to one-quarter, and direct the smith to go to that first, and if we find the foot will bear it we then proceed to the shorter mark.

The toe is always taken down first, and then the heel is lowered and, lastly, the quarters are rasped till the foot is level. By using the term "level" I do not mean to make one side of the wall the exact height from the coronary band as the other, but I sight down the axis of the limb and draw an imaginary right angle to that axis, dress the foot to that, so that when the animal stands on a level surface with feet under him the base of the feet is at right angles to the axis of the leg. By following these simple rules and making the feet in accordance, I find I am able to get all the feet in proportion and much more accurately than in any other way. If any one of the feet is found to be deformed in any way, it is always best and safest to dress it first, and then conform the others to it, so as to have a uniformity in all.

These remarks are only made to enable us to get our patients in the best possible condition, so that they may be comfortable, and if sound will probably remain so, but if unsound, and this fails to remove the unsoundness, then, of course, the animal will be in the best possible condition for operation, and we will have the satisfaction of knowing that nothing but an operation will suffice.

